# Validation of a deformable MRI to CT registration algorithm employing same day planning MRI for surrogate analysis

**DOI:** 10.1002/acm2.12296

**Published:** 2018-02-23

**Authors:** Kyle R. Padgett, Radka Stoyanova, Sara Pirozzi, Perry Johnson, Jon Piper, Nesrin Dogan, Alan Pollack

**Affiliations:** ^1^ Department of Radiation Oncology University of Miami School of Medicine Miami FL USA; ^2^ Department of Radiology University of Miami School of Medicine Miami FL USA; ^3^ MIM Software, Inc. Beachwood OH USA

**Keywords:** CT, deformable, MRI, multimodality, registration

## Abstract

**Purpose:**

Validating deformable multimodality image registrations is challenging due to intrinsic differences in signal characteristics and their spatial intensity distributions. Evaluating multimodality registrations using these spatial intensity distributions is also complicated by the fact that these metrics are often employed in the registration optimization process. This work evaluates rigid and deformable image registrations of the prostate in between diagnostic‐MRI and radiation treatment planning‐CT by utilizing a planning‐MRI after fiducial marker placement as a surrogate. The surrogate allows for the direct quantitative analysis that can be difficult in the multimodality domain.

**Methods:**

For thirteen prostate patients, T2 images were acquired at two different time points, the first several weeks prior to planning (diagnostic‐MRI) and the second on the same day as the planning‐CT (planning‐MRI). The diagnostic‐MRI was deformed to the planning‐CT utilizing a commercially available algorithm which synthesizes a deformable image registration (DIR) algorithm from local rigid registrations. The planning‐MRI provided an independent surrogate for the planning‐CT for assessing registration accuracy using image similarity metrics, including Pearson correlation and normalized mutual information (NMI). A local analysis was performed by looking only within the prostate, proximal seminal vesicles, penile bulb, and combined areas.

**Results:**

The planning‐MRI provided an excellent surrogate for the planning‐CT with residual error in fiducial alignment between the two datasets being submillimeter, 0.78 mm. DIR was superior to the rigid registration in 11 of 13 cases demonstrating a 27.37% improvement in NMI (*P* < 0.009) within a regional area surrounding the prostate and associated critical organs. Pearson correlations showed similar results, demonstrating a 13.02% improvement (*P* < 0.013).

**Conclusion:**

By utilizing the planning‐MRI as a surrogate for the planning‐CT, an independent evaluation of registration accuracy is possible. This population provides an ideal testing ground for MRI to CT DIR by obviating the need for multimodality comparisons which are inherently more challenging.

## 1 INTRODUCTION

Magnetic Resonance Imaging (MRI) has become invaluable in the management of prostate cancer because of superior visualization of soft tissues in the pelvis. For radiotherapy, this has resulted in improved staging and delineation of the dominant tumor lesion, radiation targets, and organs at risk.^1^ Effectively incorporating MRI information into the radiation treatment (RT) planning process requires robust registration tools between Computed Tomography (CT) and MRI. The Radiology and Radiation Oncology communities have expressed interest in utilizing deformable image registration (DIR) techniques in an attempt to overcome the limitations of rigid registrations.^2^, ^3^ DIR in the prostate is inherently challenging due to a variety of factors including significant variation in anatomy due to variability in rectal and bladder filling, differences in patient positioning, and incomplete knowledge and modeling of how these tissues deform over time and motion. Specifically regarding MRI to CT deformation in prostate, there are significant differences in the properties of MRI and CT imaging datasets.^2^, ^4^

For DIRs, several strategies have been developed to characterize and quantify DIR algorithms.^3^, ^5^, ^6^ This is an area of active development but several tools exist to characterize and validate DIRs, which include physical phantoms,^7^, ^8^ digital phantoms,^9^, ^10^ and anatomical landmarks for validation.^11^, ^12^ In the multimodality DIR setting, fewer validation strategies exist and creating them is even more challenging. Presented here is a novel method for evaluation of multimodality registrations of the prostate between diagnostic‐MRI and radiation treatment planning‐CT by utilizing a planning‐MRI after fiducial marker placement as a surrogate. By using the surrogate, direct quantitative analysis utilizing spatial intensity‐based metrics can be employed which otherwise would be difficult to implement in multimodality settings. The purpose of this work is to provide a novel tool for evaluation multimodality registrations. While there are existing methods for evaluating registrations between the same modalities (CT to CT, MRI to MRI), there are very few that deal with multimodality registrations.

## 2 METHODS

### 2.A Patient population

Patients were enrolled in one of two Institutional Review Board Clinical Trials for delivery of RT boost to identified multiparametric‐MRI (mpMRI)‐defined tumor lesions in the prostate: Hypofractionated External Beam Image‐Guided Highly Targeted Radiotherapy trial (NCT01411332); and Lattice Extreme Ablative Dose Radiotherapy for Prostate Cancer trial (NCT01411319). From these studies, 13 patient MRI and CT datasets where both diagnostic and planning MRI datasets were collected on the same MRI instrument were used, details are listed in Table 1.

**Table 1:** Summary of patient information (left) and discrepancy (mm) of fiducial alignment between planning‐MRI and planning‐CT (right)

Subject information summary								Plan‐MRI to plan‐CT rigid fiducial accuracy (mm)				
ID#	Age	Prostate volume (mL)	# of local alignments	Diagnostic bladder volume	Plan bladder volume	Diagnostic rectum volume	Plan rectum volume	Fiducial #1	Fiducial #2	Fiducial #3	Fiducial #4	Average
Subject #1	73	42.3	29	268	124	29	27	1.83	2.19	1.10	0.83	1.49
Subject #2	67	100.4	41	103	226	26	20	1.58	0.37	0.43	0.66	0.76
Subject #3	68	58.0	82	79	427	37	54	0.44	1.10	1.59	0.70	0.96
Subject #4	71	56.1	53	86	217	106	39	0.88	0.72	1.23	0.40	0.81
Subject #5	72	44.4	37	128	94	23	26	0.82	0.42	0.81	0.83	0.72
Subject #6	73	84.2	38	180	129	66	60	2.06	1.12	2.81	1.56	1.89
Subject #7	62	32.0	41	68	52	41	36	0.00	0.11	0.35	0.42	0.22
Subject #8	86	25.5	44	145	140	32	36	0.74	0.39	1.09	N/A	0.74
Subject #9	67	94.8	46	274	299	39	39	0.87	0.26	1.02	0.88	0.76
Subject #10	76	35.1	53	119	417	45	43	0.58	0.46	0.45	N/A	0.50
Subject #11	64	26.1	41	52	109	41	36	0.19	0.20	1.52	0.56	0.62
Subject #12	69	29.5	93	118	174	46	39	0.32	0.61	0.45	0.24	0.41
Subject #13	67	30.3	80	140	230	46	45	0.30	0.22	0.20	0.19	0.23
Average	70.4	50.7	52.2	135.3	202.8	44.4	38.5		All fiducials average (mm)			0.78
St dev	6.1	26.5	20.0	69.4	117.8	21.4	10.8		All fiducials st dev (mm)			0.60

### 2.B Imaging

All patients underwent a diagnostic mpMRI study approximately 1 month prior to radiation planning‐CT. The mpMRI includes T2‐weighted, Dynamic Contrast Enhanced (DCE) and Apparent Diffusion Coefficient datasets. All mpMRI sequences were acquired with size and spacing suitable for registration with the planning‐CT. MRI exams were carried out on a Discovery‐MR750 3T‐MRI (General Electric; Chicago, Illinois). For the purposes of this study, only the T2‐weighted sequence was used. The axial T2w‐MRI has a resolution 1.25 × 1.25 × 2.5 mm^3^, Field of View: 320 × 320 mm^2^; slice thickness; 2.5 mm; 72 slices; repetition time 5500 ms and echo time 100 ms.

The diagnostic‐MRI was used to delineate the dominant lesion(s) in the prostate and provide targets for MRI‐Ultrasound fused prostate biopsy^13^ and later to plan the RT tumor boost.^14^ During the MRI‐Ultrasound‐guided prostate biopsies, four gold fiducials were placed. These fiducials are visible on subsequent planning‐MRI and the planning‐CT.

The planning‐CT and planning‐MR imaging studies were acquired 2–4 weeks following the diagnostic‐MRI. Significant effort in patient positioning and bowel/bladder preparation was undertaken for all datasets to reduce prostate deformation. Specifically, instructions on diet were as follows: magnesium citrate taken the evening before and an enema two hours before the planning‐CT/MRI. The patients were positioned supine with legs placed in a cushion to ensure reproducible positioning of the pelvis. To determine if changes in bladder and rectum volumes impact the quality of the registrations, these structures were contoured on both diagnostic and planning MRI datasets, details are listed in Table 1. The planning‐CT was acquired from the diaphragm to mid femur at a slice thickness of 2.0 mm.

Following the planning‐CT, a planning‐MRI was collected, typically within an hour to maximize similarity between these datasets. The planning‐MRI exam consists of a T2‐weighted study, a T2* fast gradient‐echo study for visualizing the gold fiducials and several other imaging studies that are not utilized in this analysis. The T2‐weighted study is collected with identical parameters as the diagnostic‐MRI study. The T2*‐weighted study, MERGE™, is acquired with the same size and spacing to match the T2‐weighted datasets.

### 2.C Registration methods

**Figure 1:**
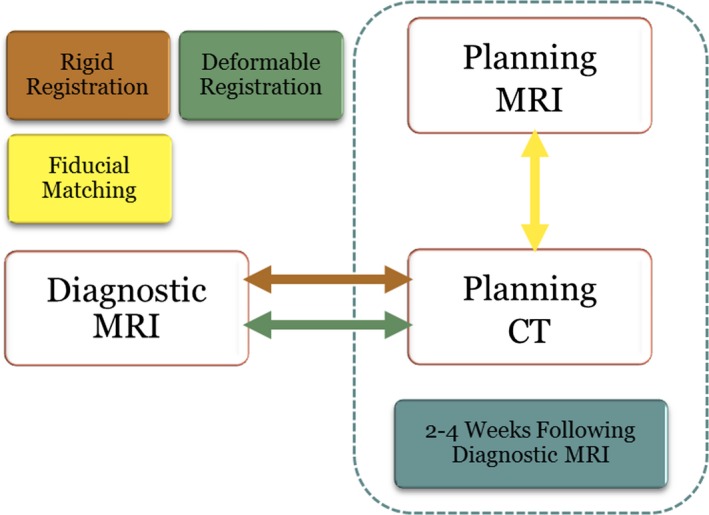
Schematic of registrations. The brown arrow indicates a rigid registration and the DIR is indicated with a green arrow. The rigid registration that employs fiducial matching is denoted with yellow arrow.

**Figure 2:**
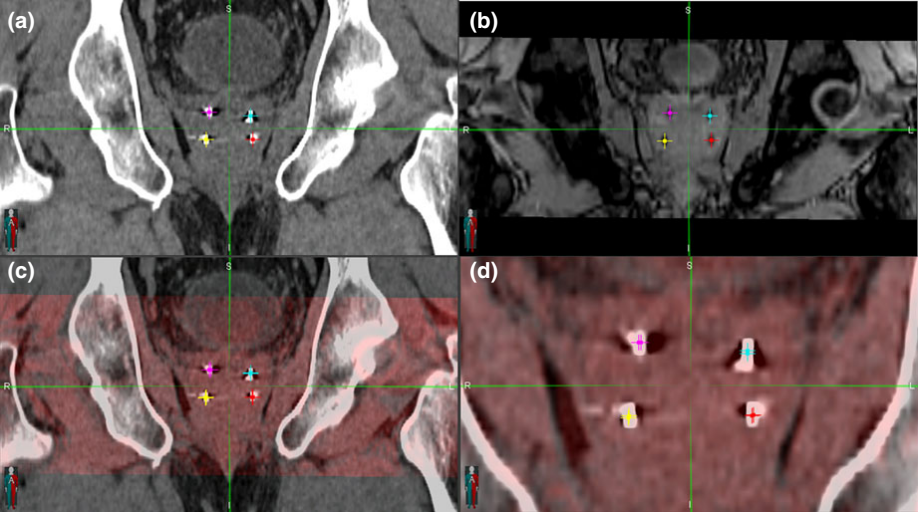
Fiducial alignment. The user‐defined location of the four fiducial markers is shown in corresponding colors on CT (a) and T2* MRI (b); (c) Alignment of the two datasets using point‐to‐point registration; (d) Enlarged view of the image in (c). demonstrating the small differences in fiducial alignment, note the two point structures can be difficult to discriminate because of their close proximity.

Rigid registrations were performed from the diagnostic‐MRI (moving) to the planning‐CT and from the planning‐MRI (moving) to the planning‐CT, as shown in Fig. 1. The diagnostic‐MRI is rigidly registered to the planning‐CT by an experienced Physicist focusing on anatomical matching; specifically balancing alignment of the prostate, penile bulb, proximal seminal vesicles, prostate/rectum interface, and prostate/bladder interface. The planning‐MRI is aligned to the planning‐CT by utilizing the four gold fiducials that are visible on both acquisitions employing a commercially available point‐based alignment method utilizing a linear least squares minimization,^15^ as shown in Fig. 2.

**Figure 3:**
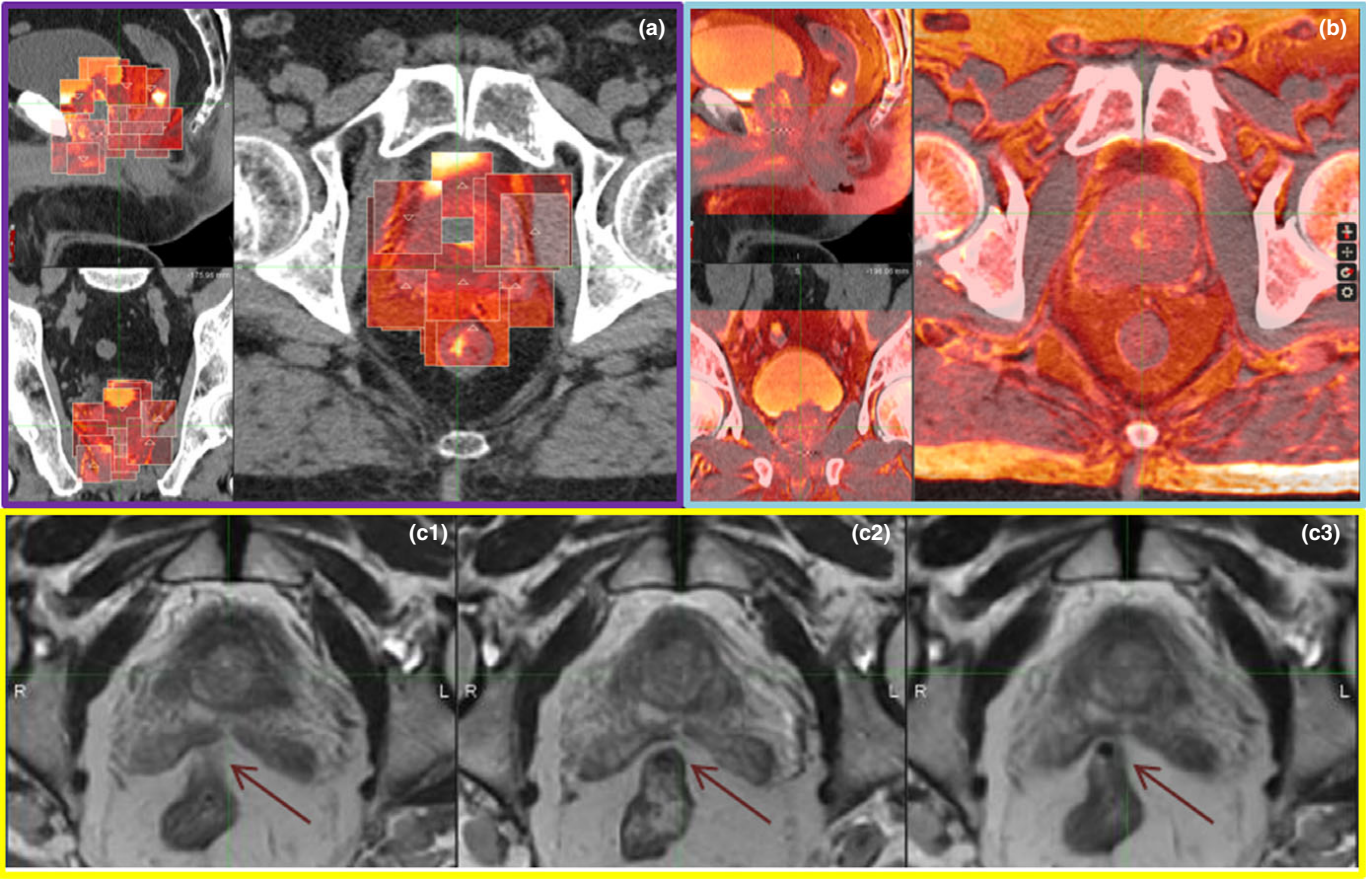
DIR procedure. (a) Local alignments used by the DIR (sagittal, coronal, and axial). MRI alignments are overlaid in red on top of the planning‐CT; (b) Resulting deformed MRI dataset blended with the planning‐CT; (c) diagnostic‐MRI(c1), planning‐MRI(c2) and deformed diagnostic‐MRI(c3). Note how the rectum in the deformed diagnostic‐MRI(c3) correlates well with the planning‐MRI(c2).

The diagnostic‐MRI was deformed to the planning‐CT utilizing a commercially available algorithm (MIM version6, MIM Software Inc.; Beachwood, OH) which synthesizes a DIR from local rigid registrations using a Gaussian mixing model to spatially weight the contributions of each alignment, no image similarity metrics are employed by the DIR. Deforming the diagnostic‐MRI to the planning‐CT begins with a rigid alignment over the area of interest. Following this procedure, manual local registrations were obtained between the two datasets by aligning key points throughout the areas of interest, which provided initial conditions for the DIR algorithm, as shown in Fig. 3. Several local alignments are distributed to ensure an accurate registration including: bony anatomy, penile bulb, prostate/bladder interface, prostate/rectum interface, lateral aspects of the prostate, and seminal vesicles, there were 52 ± 20 local alignments per patient. The number of alignments employed varied from patient to patient based upon the needs of the registration, as an example for patients where the rectum was significantly different in volume between the two studies, more local alignments would be needed in this area to achieve an acceptable registration. Deformation vector fields (DVF) were computed at each image voxel as the distance‐weighted sum of the vectors implied by each local rigid registration.

### 2.D Registration evaluation and statistics

The T2‐weighted acquisition from the planning‐MRI study was employed as the surrogate for the planning‐CT for analysis of both the rigid and DIR of the diagnostic‐MRI to the planning‐CT. Agreement between the two MRI datasets was scored using intensity‐based metrics including Pearson correlation and normalized mutual information (NMI) and significance was determined by utilizing a Student's *t*‐test with a 95% confidence limit. Pearson correlation is an often used same‐modality similarity metric which is appropriate because the diagnostic and planning images are acquired with the same protocol. NMI is also computed because of its widespread use as a metric for registration optimization and evaluation.^16^, ^17^ Following contouring of relevant anatomy by a Radiation Oncologist, a local analysis was performed in the prostate, proximal seminal vesicles, penile bulb, combined structure (consisting of all the structures combined), and the expanded structure (the combined structure expanded by 5 mm). The combined and expanded structures were included for determination of regional registration quality.

## 3 RESULTS

Alignment of planning‐MRI to planning‐CT was confirmed to be submillimeter by measuring the residual error of fiducial location between the planning‐MRI and the planning‐CT. This important result demonstrates that the planning‐MRI may be used as a surrogate for the planning‐CT when evaluating the diagnostic‐MRI to planning‐CT deformable registration. The average residual error after fiducial registration was 0.78 mm ± 0.60 mm for the 13 patients, full results are shown in Table 1.

Consistent findings were made across all comparisons with the DIRs demonstrating substantial improvements over rigid registrations. This difference was significant across all structures studied in 11 out of 13 patients utilizing both NMI and Pearson correlations. Utilizing the NMI, the DIRs were superior to the rigid registrations demonstrating a 27.37% improvement (*P*‐value 0.009) within the expanded area, similar findings were found with the Pearson correlation. Figure 3 shows an example of the conformity of the anatomy between the planning‐CT and the deformed MRI. The penile bulb had the largest improvement with the DIR resulting in gains in 12 of 13 patients when using the NMI metric, 45.17% improvement averaged over patients, and in 11 of 13 patients using the Pearson Correlation metric, 37.84% improvement. The prostate also showed gains when using the DIR with an average 27.63% improvement for NMI over rigid in 10 of 13 patients and 19.46% improvement for Pearson correlation in 11 of 13 patients. Lastly, DIR of the proximal seminal vesicles resulted in an average of 24.11% improvement in NMI over rigid in 11 of 13 patients and an average of 20.00% improvement in Pearson correlations for 12 of 13 patients, full results shown in Table 2.

**Table 2:** Results of both rigid and DIRs in the expanded contour are summarized in the upper portion of this table with 11 out of 13 patients showing higher agreement with DIR. The results for each structure averaged across all patients are shown in the lower section, *significant difference

Normalized mutual information (expanded)				Pearson correlation (expanded)		
ID#	Rigid	Deform	% Change	Rigid	Deform	% Change
Rigid and deformable registration results for each subject						
Subject #1	0.076	0.095	25.00%	0.609	0.682	11.99%
Subject #2	0.065	0.069	6.15%	0.609	0.624	2.46%
Subject #3	0.041	0.050	21.95%	0.422	0.459	8.77%
Subject #4	0.070	0.088	25.71%	0.541	0.600	10.91%
Subject #5	0.035	0.045	28.57%	0.436	0.381	−12.61%
Subject #6	0.101	0.116	14.85%	0.664	0.719	8.28%
Subject #7	0.152	0.150	−1.32%	0.777	0.801	3.09%
Subject #8	0.081	0.099	22.22%	0.641	0.731	14.04%
Subject #9	0.056	0.069	23.21%	0.603	0.656	8.79%
Subject #10	0.101	0.121	19.80%	0.696	0.763	9.63%
Subject #11	0.058	0.055	−5.17%	0.572	0.565	−1.22%
Subject #12	0.051	0.122	139.22%	0.516	0.735	42.44%
Subject #13	0.052	0.121	132.69%	0.493	0.748	51.72%

Normalized mutual information					Pearson correlation			
Structure	Rigid‐mean	Deform‐mean	% Change	*P*‐value	Rigid‐mean	Deform‐mean	% Change	*P*‐value
Rigid and deformable registration results for each structure								
Penile bulb	0.076	0.111	45.17%	0.000*	0.473	0.652	37.84%	0.002*
Prostate	0.076	0.097	27.63%	0.032*	0.555	0.663	19.46%	0.022*
Prox SV	0.048	0.059	24.11%	0.042*	0.370	0.444	20.00%	0.057
Combined	0.072	0.093	30.00%	0.014*	0.557	0.654	17.41%	0.008*
Expanded	0.072	0.092	27.37%	0.009*	0.576	0.651	13.02%	0.013*

To investigate regional results of the registrations, the combined and expanded structures were implemented. The combined structure had 10 out of 13 patients with improved NMI, 30.0% average, with similar results for Pearson correlations. Similarly, the expanded structure had 11 of 13 patients with improved NMI, 27.37% average, with similar results for Pearson correlations. Changes in bladder and rectal volumes between the diagnostic and planning datasets did not correlate with NMI or Pearson metrics for either rigid or DIR registrations. To summarize, the deformable registrations were superior to the rigid registrations in all structures and significantly better in all but the proximal seminal vesicles.

## 4 DISCUSSION

**Figure 4:**
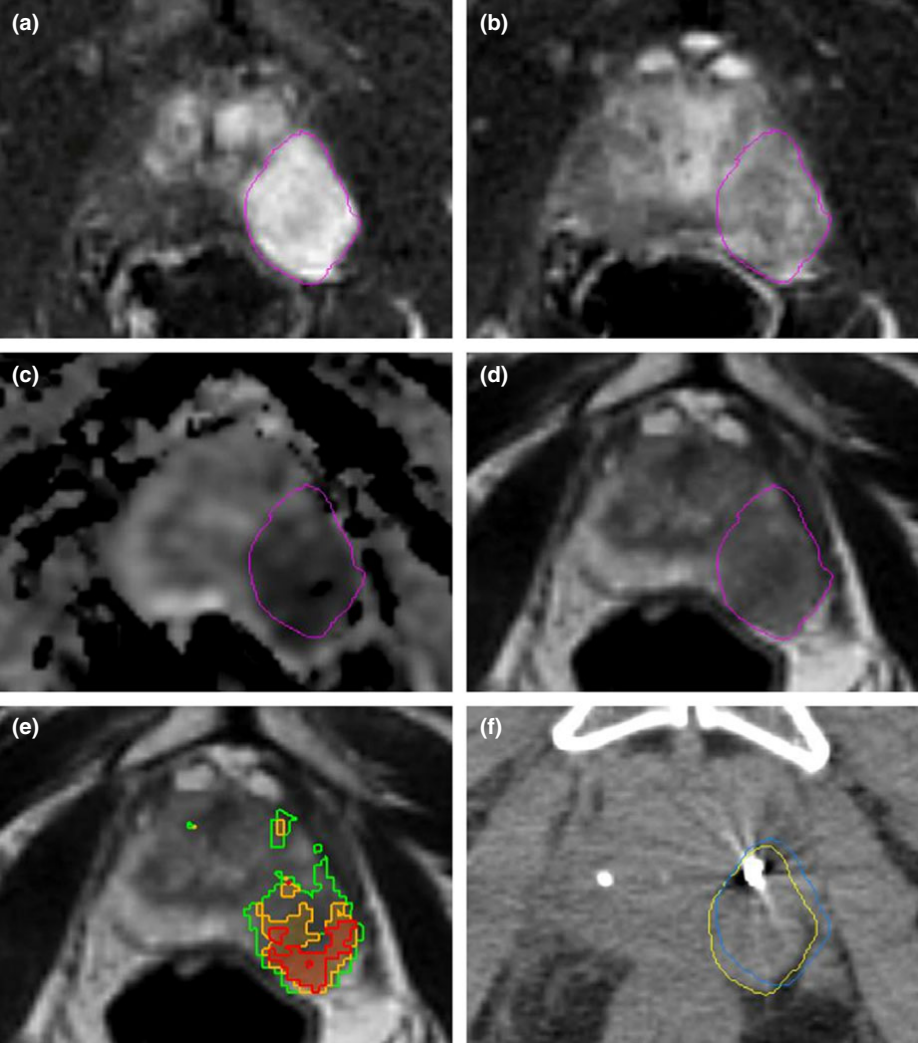
DIR and GTV delineation on mpMRI. (a) Early phase of DCE‐MRI; (b) Late phase of DCE‐MRI; (c) Apparent diffusion coefficient map; (d) T2‐image showing the physician defined dominant tumor, GTV; (e) Probability map of dominant tumor location based upon analysis of mpMRI; (f) (planning CT) Physician defined dominant lesion has been transferred to the CT using a rigid registration (blue) and a deformable registration (yellow). Note how the contour transferred using the DIR follows the anatomy of the CT scan more closely than the rigidly transferred contour.

CT to CT DIRs are increasingly being relied upon to map crucial information between datasets. The ability to extend these DIRs into the multimodality setting would be enthusiastically received by the radiation oncology community, if it can be done with accuracy and confidence. Currently, MRI is heavily utilized to aid in the contouring of target and OAR volumes on planning CT studies.^1^, ^4^ Increasing the precision of MRI to CT registrations with DIR techniques will improve contouring which may result in better target coverage and/or OAR sparing.^2^ A novel and currently actively researched application that would greatly benefit from accurate MRI to CT deformable registrations is mapping of prostate subvolumes identified utilizing mpMRI as the dominant lesion/s to be used as radiation boost targets, as shown in Fig. 4.^14^, ^18^ MRI to CT deformable registration is an attractive way to link these areas between image sets.

The characterization of multimodality rigid and DIRs in this study rely on the planning‐MRI's ability to act as a surrogate for the planning‐CT, thus allowing MRI to MRI comparisons to be made. Due to the fiducials being visible on both datasets, the short time between the two acquisitions, the bowel and bladder preparation and the reproducible positioning of the patient, the registration between the planning‐MRI and planning‐CT is of exceptional quality; less than 1 mm on average, this is demonstrated in Table 1 and Fig. 2. This attention to bowel and bladder preparation may be why no correlation was found between a change in bladder/rectal volume between diagnostic and planning acquisitions and registration performance.

By review of the registrations and by the correlative metrics determined by the study, both rigid and DIRs were of high quality. The DIRs resulted in higher correlation metrics than the rigid registrations, but the rigid registrations were also well‐matched likely due to the focus on reproducible patient setup. For the rigid registrations, there was variability in the correlation metrics for different structures with the penile bulb and the proximal seminal vesicles having the lowest correlations. This may be explained by noting that the prostate was the focus of the rigid registrations and that the registration quality of the bulb and the seminal vesicles are considered to a lesser extent than the prostate. The increased accuracy of the deformable registrations may be explained by the rigid registration only being able to optimize the registration on a small anatomical area. However, improvements were also noted within the prostate with DIR. While prostate is the focus of the work presented here, other body sites may also benefit from this approach. Specifically, sites where MRI is often incorporated into the treatment planning process and where rigid registrations are frequently suboptimal; abdomen, head and neck, brain pre/postsurgery, etc.

The DIR validation technique described in this manuscript has several unique attributes. The data employed were collected from protocol patients and do not use simulated images or images of artificial materials. This has the benefit of testing the DIR algorithm using images collected on humans using the equipment present in the clinic, thus reflecting the clinic workflow. Another advantage is that none of the datasets are simulated or altered prior to DIR, thus eliminating any potential issues of utilizing artificial materials or simulated data. One challenge with this validation technique is that it lacks a known DVF to compare the resulting deformable registration to and instead relies on correlations between datasets. While a known DVF is a robust solution, the correlation metrics implemented here share the ability to evaluate registration accuracy across any region that is defined by the user, albeit not pixel by pixel. The emergence of multimodality image deformable registrations holds great promise and will facilitate a more seamless integration of MRI and other imaging modalities into the RT planning process among other applications outside of radiation oncology.

In order for multimodality DIRs to be widely adopted, robust validation of these algorithms is necessary. This unique method of validation of multimodality registration utilizing a planning‐MRI as a surrogate complements existing validation methods.

## CONFLICT OF INTEREST

Sara Pirozzi and Jon Piper are both employees of MIM Software Inc., which created the deformable algorithm used in this work. Jon Piper also has an ownership interest in MIM Software Inc.

## References

[1] Villeirs GM , L Verstraete K , De Neve WJ , De Meerleer GO . Magnetic resonance imaging anatomy of the prostate and periprostatic area: a guide for radiotherapists. Radiother Oncol. 2005;76:99–106. 16023234 10.1016/j.radonc.2005.06.015

[2] Brock KK , Deformable Registration Accuracy C . Results of a multi‐institution deformable registration accuracy study (midras). Int J Radiat Oncol Biol Phys 2010;76:583–596. 19910137 10.1016/j.ijrobp.2009.06.031

[3] Kashani R , Hub M , Balter JM , et al. Objective assessment of deformable image registration in radiotherapy: a multi‐institution study. Med Phys. 2008;35:5944–5953. 19175149 10.1118/1.3013563 PMC2673610

[4] Rasch C , Barillot I , Remeijer P , Touw A , van Herk M , Lebesque JV . Definition of the prostate in ct and mri: a multi‐observer study. Int J Radiat Oncol Biol Phys. 1999;43:57–66. 9989514 10.1016/s0360-3016(98)00351-4

[5] Varadhan R , Karangelis G , Krishnan K , Hui S . A framework for deformable image registration validation in radiotherapy clinical applications. J Appl Clin Med Phys. 2013;14:192–213. 10.1120/jacmp.v14i1.4066 PMC3732001 23318394

[6] Wang H , Dong L , O'Daniel J , et al. Validation of an accelerated ‘demons’ algorithm for deformable image registration in radiation therapy. Phys Med Biol. 2005;50:2887–2905. 15930609 10.1088/0031-9155/50/12/011

[7] Singhrao K , Kirby N , Pouliot J . A three‐dimensional head‐and‐neck phantom for validation of multimodality deformable image registration for adaptive radiotherapy. Med Phys. 2014;41:121709. 25471956 10.1118/1.4901523

[8] Sun J , Dowling J , Pichler P , et al. Mri simulation: end‐to‐end testing for prostate radiation therapy using geometric pelvic mri phantoms. Phys Med Biol. 2015;60:3097–3109. 25803177 10.1088/0031-9155/60/8/3097

[9] Pukala J , Meeks SL , Staton RJ , Bova FJ , Mañon RR , Langen KM . A virtual phantom library for the quantification of deformable image registration uncertainties in patients with cancers of the head and neck. Med Phys. 2013;40:111703. 24320411 10.1118/1.4823467

[10] Nie K , Chuang C , Kirby N , Braunstein S , Pouliot J . Site‐specific deformable imaging registration algorithm selection using patient‐based simulated deformations. Med Phys. 2013;40:041911. 23556905 10.1118/1.4793723

[11] Vandemeulebroucke J , Sarrut D , Clarysse P . The popi‐model, a point‐validated pixel‐based breathing thorax model. XVth international conference on the use of computers in radiation therapy (ICCR). Vol. 2; Citeseer; 2007: 195–199.

[12] Kaus MR , Brock KK , Pekar V , Dawson LA , Nichol AM , Jaffray DA . Assessment of a model‐based deformable image registration approach for radiation therapy planning. Int J Radiat Oncol. 2007;68:572–580. 10.1016/j.ijrobp.2007.01.056 17498570

[13] Sandler K , Patel M , Lynne C , et al. Multiparametric‐mri and targeted biopsies in the management of prostate cancer patients on active surveillance. Front Oncol. 2015;5:1–4. 25674540 10.3389/fonc.2015.00004 PMC4306300

[14] Bossart EL , Stoyanova R , Sandler K , et al. Feasibility and initial dosimetric findings for a randomized trial using dose‐painted multiparametric magnetic resonance imaging‐defined targets in prostate cancer. Int J Radiat Oncol Biol Phys. 2016;95:827–834. 27020109 10.1016/j.ijrobp.2016.01.052

[15] Huisman HJ , Fütterer JJ , van Lin EN , et al. Prostate cancer: precision of integrating functional mr imaging with radiation therapy treatment by using fiducial gold markers. Radiology. 2005;236:311–317. 15983070 10.1148/radiol.2361040560

[16] Maes F , Collignon A , Vandermeulen D , Marchal G , Suetens P . Multimodality image registration by maximization of mutual information. IEEE Trans Med Imaging. 1997;16:187–198. 9101328 10.1109/42.563664

[17] Veninga T , Huisman H , van der Maazen RW , Huizenga H . Clinical validation of the normalized mutual information method for registration of ct and mr images in radiotherapy of brain tumors. J Appl Clin Med Phys. 2004;5:66–79. 15753941 10.1120/jacmp.v5i3.1959 PMC5723487

[18] Kuban DA , Levy LB , Cheung MR , et al. Long‐term failure patterns and survival in a randomized dose‐escalation trial for prostate cancer. Who dies of disease? Int J Radiat Oncol Biol Phys. 2011;79:1310–1317. 20493642 10.1016/j.ijrobp.2010.01.006

